# Melanopsin Bistability: A Fly's Eye Technology in the Human Retina

**DOI:** 10.1371/journal.pone.0005991

**Published:** 2009-06-24

**Authors:** Ludovic S. Mure, Pierre-Loic Cornut, Camille Rieux, Elise Drouyer, Philippe Denis, Claude Gronfier, Howard M. Cooper

**Affiliations:** 1 Department of Chronobiology, INSERM, U846, Stem Cell and Brain Research Institute, Bron, France; 2 University of Lyon, Lyon I, UMR-S 846, Lyon, France; 3 Department of Ophthalmology, CHU de Lyon Hopital Edouard Herriot, Lyon, France; Tufts University, United States of America

## Abstract

In addition to rods and cones, the human retina contains light-sensitive ganglion cells that express melanopsin, a photopigment with signal transduction mechanisms similar to that of invertebrate rhabdomeric photopigments (IRP). Like fly rhodopsins, melanopsin acts as a dual-state photosensitive flip-flop in which light drives both phototransduction responses and chromophore photoregeneration that bestows independence from the retinoid cycle required by rods and cones to regenerate photoresponsiveness following bleaching by light. To explore the hypothesis that melanopsin in humans expresses the properties of a bistable photopigment *in vivo* we used the pupillary light reflex (PLR) as a tool but with methods designed to study invertebrate photoreceptors. We show that the pupil only attains a fully stabilized state of constriction after several minutes of light exposure, a feature that is consistent with typical IRP photoequilibrium spectra. We further demonstrate that previous exposure to long wavelength light increases, while short wavelength light decreases the amplitude of pupil constriction, a fundamental property of IRP difference spectra. Modelling these responses to invertebrate photopigment templates yields two putative spectra for the underlying R and M photopigment states with peaks at 481 nm and 587 nm respectively. Furthermore, this bistable mechanism may confer a novel form of “photic memory” since information of prior light conditions is retained and shapes subsequent responses to light. These results suggest that the human retina exploits fly-like photoreceptive mechanisms that are potentially important for the modulation of non-visual responses to light and highlights the ubiquitous nature of photoswitchable photosensors across living organisms.

## Introduction

The human retina contains two phylogenetically distinct classes of photoreceptors [1]. Rods and cones are transducin mediated ciliary photoreceptors typical of vertebrates, while melanopsin, contained in a subset of light-sensitive retinal ganglion cells (mRGCs) shares structural homology and signal transduction mechanisms involving Gq-type G protein of invertebrate rhabdomeric photopigments (IRP) [2], [3], [4], [5], [6] such as fly rhodopsins. IRPs are bistable and possess two photosensitive states in which photon absorption at one wavelength initiates the phototransduction cascade of the photopigment bound *11-cis-*retinal (rhodopsin - R state) while subsequent absorption at a second wavelength photoisomerizes *all-trans-retinal* (metarhodopsin - M state) back to *11-cis* to restore responsiveness [7]. This property translates to photoreception in IRPs that is independent from specialized extrinsic chromophore regeneration processes (i.e; retinoid cycle of rods and cones), can be modulated by previous light exposure that drives the photopigment between the two R and M isoforms and includes a prolonged depolarisation after potential (PDA; [8], [9]). Like IRPs, melanopsin expressed *in vitro* also displays a light dependent inter-convertibility between two spectral absorption states that is driven by exposures to short and long wavelength light [3], [4], [10], [11]. This capacity for bistablility has been proposed to explain the sustained photoresponses and resistance to bleaching of mRGCs [12], [13], [14], [15] and of SCN neurons [12], [16] as well as the ability of the pupil to maintain constriction in continuous bright light [12], [17].

We assayed the pupillary light reflex (PLR) in humans as a tool to explore the hypothesis that melanopsin expresses the properties of a bistable photopigment *in vivo*, but applied a light stimulation strategy and mathematical models originally designed to demonstrate bistability and define photopigment states in invertebrates [18], [19], [20]. We analysed a novel component of the pupil response that displays the hallmark properties of melanopsin-driven photoresponses (spectral, sustained and persistent responses) that in animal models are eliminated following genetic invalidation of the Opn4 photopigment [12], [17], [21], [22]. To confirm the hypothesis of melanopsin bistability in the human retina, three diagnostic features of IRP photopigment systems were required: (1) that the system can be driven to a stable state of equilibrium, dependent on stimulus exposure conditions, (2) that a difference spectrum can be generated that induces increases or decreases in responsiveness according to previous adapting light exposure conditions and (3) that IRP models of the equilibrium and difference spectra derive two underlying photopigment states of the bistable system that are consistent with known *in vitro* spectral absorbance of expressed melanopsin pigment.

## Materials and Methods

### Subjects

Twelve subjects with normal color vision participated in this study (5 males and 7 females ranging from 24 to 30 years). All experiments were performed in accordance with Institutional guidelines and the research protocol received the necessary ethical approvals (RBM C06-17). Written informed consent was obtained from each subject prior to inclusion in the experiment. Subjects were examined for normal color vision, visual field and verified that there was no detectable ophthalmic problems. Subjects were emmetropic and none were under medication during the testing period. All experiments were performed between 9:00 and 13:00 hrs, subjects were tested only once per day and had at least one day free between 2 consecutive sessions. All the experiments were conducted between Jan 10 to March 4. For a given subject testing was completed within 36 days maximum. In between sessions subjects returned to their normal occupations. Subjects also filled in 3 questionnaires and had normal chronotypes (Horne and Ostberg questionnaire, Munich chronotype questionnaire) and sleep quality (Pittsburg Sleep Quality Index, PSQI).

### Pupil Measures

Monochromatic light stimulations were used to record consensual pupillary constriction responses. The pupil size was recorded from the unilluminated eye using an infrared video pupil tracking system (ViewPoint, Arrington). A video camera and an infrared illuminator were set up in a plane parallel to the cornea. Behind the recording system, a small LED was located to allow the subject to keep a stable gaze on the same point during the entire experiment. Pupil aperture size measures are based on an on-line contrast detection between the pupil and the iris and an algorithm that permits to track circularity of the pupil. Data, pupil area and circularity coefficient are collected at a sampling rate of 30 images/sec.

### Light stimulation

Monochromatic stimulation was produced using a tungsten halogen light source (24 V–150 W Eke bulb) with heat absorbing, collimating lenses and Schott interference filters (10 nm half band width). Light was projected through an opal diffuser providing a uniform, pattern-less stimulus that encompassed the entire visual field of one eye. Stimulus irradiance and duration was controlled with an electronic shutter (Uniblitz) under a computer program. Irradiance levels and spectral distribution were monitored with a photometer (International Light IL700) and spectrophotometer (Ocean Optics S4000). The pupil of the stimulated eye was dilated by topical application of tropicamid on the cornea.

### Protocol and Data analysis

#### General

Data are analyzed using Matlab (Mathworks©). Artifacts in pupil measures, for example due to eye movements or eye blinks were eliminated using the circularity coefficient of the pupil outline to detect absence of fixation. Sample sequences containing more than 10% deviation from circularity were excluded from the analysis. To correct for individual variations in pupil size, data were normalized for each subject to mean pupil area measured during the last 5 minutes of dark adaptation prior to light exposures. Pupil data were smoothed using a non-parametric locally weighted linear regression (Lowess).

#### Kinetics and spectral sensitivity of the PLR

To establish initial criteria of pupil kinetics and sensitivity, two of the subjects were tested using 10 wavelengths (400, 420, 440, 460, 480, 500, 530, 560, 590, 620 nm) at 6 different irradiances (1e10, 1e11, 1e12, 1e13, 1e14 and 3e14 photons/cm^2^/sec). For all the experiments, the irradiances were corrected for lens absorbance based on data of Stockman et al [23]. In this phase, each stimulation lasted 5 minutes and was preceded by 40 minutes of complete darkness to allow dark adaptation of rods and cones.

Different temporal sequences of the pupillary response were analyzed during several time windows of the initial pupillary constriction between 0–30 seconds after lights-on (phasic and tonic responses [15], [24]. The steady-state of pupil constriction was measured during the prolonged constant light exposure (3–5 minutes). Finally, post-stimulus persistence of pupillary constriction was analyzed during the first minute after lights-off.

The relation between irradiance and pupillo-constriction was fit using the Hill equation as follows: 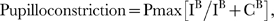
where I is irradiance, Pmax is maximal pupillo-constriction, B is a constant, and C is the irradiance at which half-maximal pupillo-constriction is produced. B and C were varied to obtain best fits. We then generated plots of relative quantum sensitivity of different temporal phases of the response defined above as a function of wavelength to establish pupil spectral sensitivity.

#### Effects of prior light exposure on the PLR

To assay the effects of prior light exposure we used procedures previously applied to studies of invertebrate photoreception [9], [18], [19] and in the mouse [12]. Ten additional subjects were initially tested. The subjects first remained in darkness for 40 minutes before receiving an initial 480 nm light stimulation of 5 minutes (1e12 photons/cm^2^/sec; reference stimulus) and after a second period of 50 minutes in darkness they received a second, identical 480 nm light stimulation (test stimulus) of 5 minutes. Within the intervening period of darkness an adapting pre-exposure stimulus of different wavelengths (620 nm: n = 10; 480 nm; n = 5; black: n = 5) was administered 5 minutes after the initial reference stimulus for a duration of 5 minutes. Results are expressed as the mean variation in pupil constriction between the first 480 nm reference stimulation and the subsequent 480 nm test stimulation.

The effects on pupil constriction of adapting pre-exposures across the entire visual spectrum using 11 different wavelengths (400, 420, 440, 460, 480, 500, 530, 560, 590, 620, 650 nm) were explored in 4 subjects (chosen for their ability to maintain fixation during the long exposure sequences). We fit the resulting *difference spectrum* [9], [19], [20] using a 5-degree polynomial curve to obtain the best fit.

The *equilibrium spectrum* [9], [19] for the steady-state pupil constriction (n = 4 subjects) is based on measures obtained in each subject at each of the 11 different pre-exposition wavelengths and corrected according to the complete irradiance dose response curves of the 2 subjects recorded in the initial experiments to obtain the half saturation values.

#### Calculation of R and M spectra

To determine the putative R and M peaks we used Stavenga's mathematical method [19] that was originally developed for spectrophotometric data but subsequently adapted for use with *in vivo* measurements (see [9]). In brief, Stavenga's method is based on use of combined measures of equilibrium and difference spectra to derive the underlying the R and M absorption spectra using the following formulas: 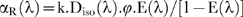


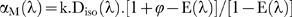
where α is the absorption, D the difference and E the equilibrium spectra, all are a function of wavelength λ. k is a constant related to *in vitro* parameters (concentrations, sample thickness, transmittance, etc [19]) here set to a value of 1.0. The relative quantum efficiency, ϕ, is generally unknown and in this case is set to 1.0 [9]. Note that this last constant only affects the relative amplitudes of the R and M peaks but not the position of the spectral peak or shape of the spectra that are constrained by the *retinal* nomogram function [19].

In this study we used 480 nm light as the test and reference stimuli since this has been reported as the peak wavelength of sensitivity for melanopsin expressed *in vitro* [3], [10], [11], [25], and for retinal mRGCs recordings [26], [27], [28]. Thus, the isosbestic point was determined after the experiment. As a consequence, we use D_480_ instead of D_iso_ but this does not influence the calculated values of the positive and negative peaks but may slightly effect the relative amplitudes of the peaks [19], [20].

All numerical values are expressed as mean±SEM.

## Results

### Pupillary constriction to light is composed of distinct temporal response components

**Figure 1:**
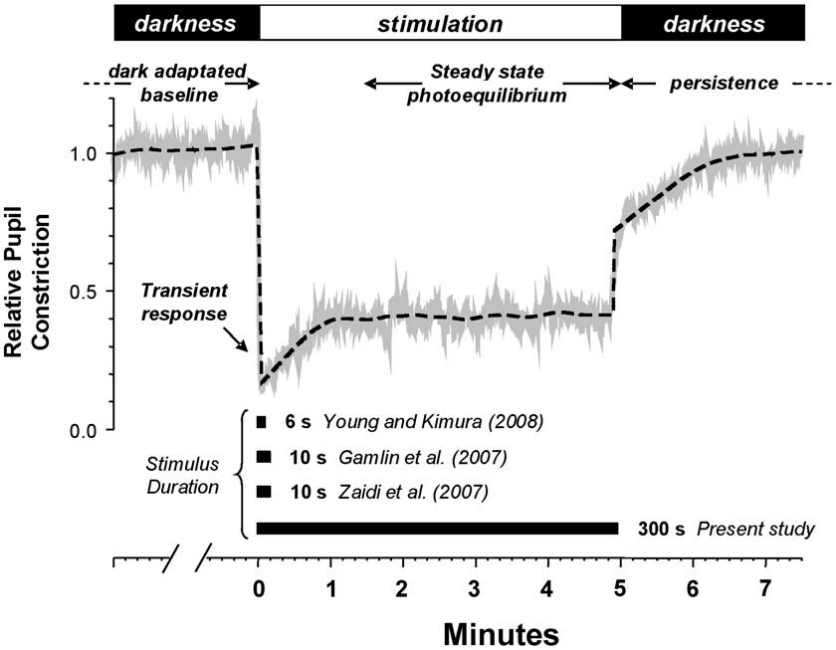
The pupillary light reflex response is composed of several different temporal components. In a totally dark-adapted (40 min) subject, the consensual pupil response was measured in one eye while illuminating the entire visual field of the opposite eye (1e13 photons/cm^2^/sec at 480 nm). At stimulus onset the pupil rapidly constricts to a minimal size referred to as the *transient* response that lasts 500–1000 ms [15], [24]. This is followed by a redilation to a *tonic* or *sustained* pupil size, previously described for durations <10 sec (bars in bottom of figure; [15], [29], [37]). By maintaining a prolonged constant illumination of the retina, pupil size stabilizes to a true *steady state “photoequilibrium*”. The time required to attain this photoequilibrium depends on the irradiance and wavelength of the stimulus. In the case shown the pupil reaches stable constriction in roughly 1 minute and maintains that constriction level until extinction of the stimulus. At light offset, the pupil slowly recovers to the dilated dark adapted state (*post-stimulus persistence*). The solid gray line shows the raw pupil constriction recordings and the dashed line the smoothed response function (further details in Methods).

The pupillary light reflex (PLR) in humans and primates is composed of an initial, rapid pupil constriction followed by a sustained constriction and, after light extinction, a post-stimulus persistence of pupil constriction [15], [24], [29]. However, the kinetics of the PLR in humans has not been fully described, as previous studies in primate and non-primate mammals have rarely investigated stimulus durations longer than 15 seconds [15], [24], [29], [30] whereas the pupil normally copes with continuous light exposure. Although exposure to very intense light can rapidly lead to a sustained pupil response, by use of very long duration light exposures, we find that for the majority of wavelengths and irradiances tested (∼90%) a true stabilized state of pupil constriction is only attained within 3 minutes (199.4±2.5 sec for the remaining ∼10%). Here, we define this constant pupillo-constriction as the “*steady state equilibrium*” **(** **figure 1** **)** for which the linear regression of the response deviates less than 5% from horizontal. Thus for the definition and analysis of this parameter we used the mean pupil size measured between 3–5 minutes. Once attained, the *state of equilibrium* persists unchanged until extinction of the light stimulus (up to 10 min, data not shown), after which the pupil slowly returns (post-stimulus persistence; **figure 1**) to the dark adapted baseline condition.

**Figure 2:**
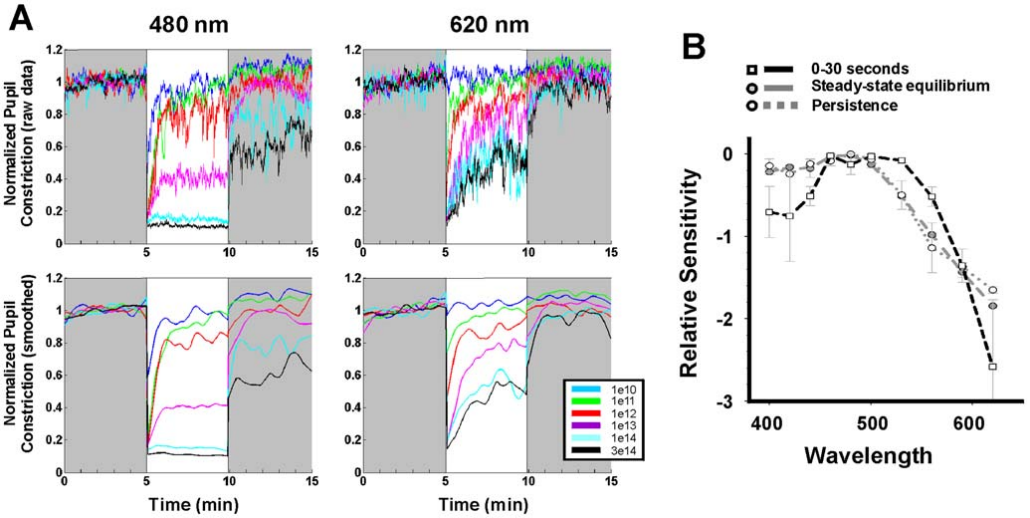
Spectral responses of the pupillary light reflex. (A) Comparison of the PLR to 480 nm and to 620 nm monochromatic long duration (5 min) stimulations at 6 different irradiances in a single subject. The plates above illustrate the raw pupil data and below the smoothed response. Following the initial pupil constriction, the steady state equilibrium and the persistent responses are observable in all but the lowest irradiances and depend on wavelength and light intensity. The amplitude of the steady state equilibrium response is rapidly attained and particularly robust at 480 nm for the highest irradiances used. The persistent responses are also greater at 480 nm compared to 620 nm at equivalent irradiances (see also figure S2). Note that for the higher irradiances (>1e13 photons/cm^2^/sec) the pupil has not yet returned to the baseline within 5 min after extinction of the stimulus. (B) Action spectrum of the different components of PLR obtained from the intensity dose response curves (as illustrated in A) from 2 subjects. The action spectrum for the first 30 seconds of the PLR (black dashed line) shows broad sensitivity over the blue-green region (460–530 nm). This curve is similar to the spectra obtained between 0–5, 5–10 and 5–30 sec (figure S1) and to data in humans and primates [15], [29]. This profile differs from the action spectrum for the steady state equilibrium and the persistence response functions (respectively the gray dashed and gray dotted lines) that are virtually identical and show maximal sensitivity in the short wavelength region (460–480 nm).

We assayed the spectral responses of the PLR by analysing response profiles sampled during different temporal windows of light exposure. It has long been recognized that the spectral response of the early PLR in humans is driven by multiple photoreceptors since the action spectrum does not fit the absorbance template of a single retinal_1_-based photopigment and that the relative contributions of rods, cones and melanopsin evolve over the duration of light exposure [15], [24], [29], [30]. The PLR analysed at several time windows of the initial 30 sec of exposure (**figure 2B** **, figure S1**) yields a family of curves with similar response profiles and a broad region of sensitivity from 460–530 nm in agreement with previous studies [15], [29], [30]. In contrast to the early PLR, the spectral response profiles for the steady-state equilibrium PLR and for the post-stimulus persistent responses are indistinguishable from each other and show maximal sensitivity in the blue region of the spectrum (approximately 460–480 nm; **figure 2B** **, figure S1**).

### Steady state equilibrium and post-stimulus pupil responses to light

Gamlin et al. [15] have previously demonstrated that the spectral response of the post-stimulus pupillo-constriction is consistent with a melanopsin driven photoresponse and that the responses are equivalent under either normal conditions or with pharmacological blockade of rod and cone inputs. The similarity of the long-term steady state equilibrium we define in humans and the post-stimulus response functions strongly argues in favor of a common melanopsin-driven origin. The dependence on melanopsin is further confirmed by observations that in mice lacking melanopsin, the ability to sustain pupil constriction and SCN neuronal firing to bright light, as well as the post–stimulus responses after light extinction are absent [12], [17]. This post-stimulus persistence of the pupil, also observed in mRGCs [26], [27], [28] and SCN neuron photoresponses [16], is proposed to correspond to the prolonged depolarizing after potential (PDA) of IRPs [8], [13] which in fly rhodopsin can persist for several minutes to hours [9]. The post-stimulus constriction of the human pupil can also persist for several minutes (>5 min) depending on the wavelength and irradiance of the preceding light stimulus (**figure S2**). The action spectra of the post-stimulus response based on response amplitude and on the relative duration required to return to the dark adapted state (data not shown) are similar and comparable with the action spectra for the steady state equilibrium.

### Prior light exposure alters subsequent pupil responses to light

**Figure 3:**
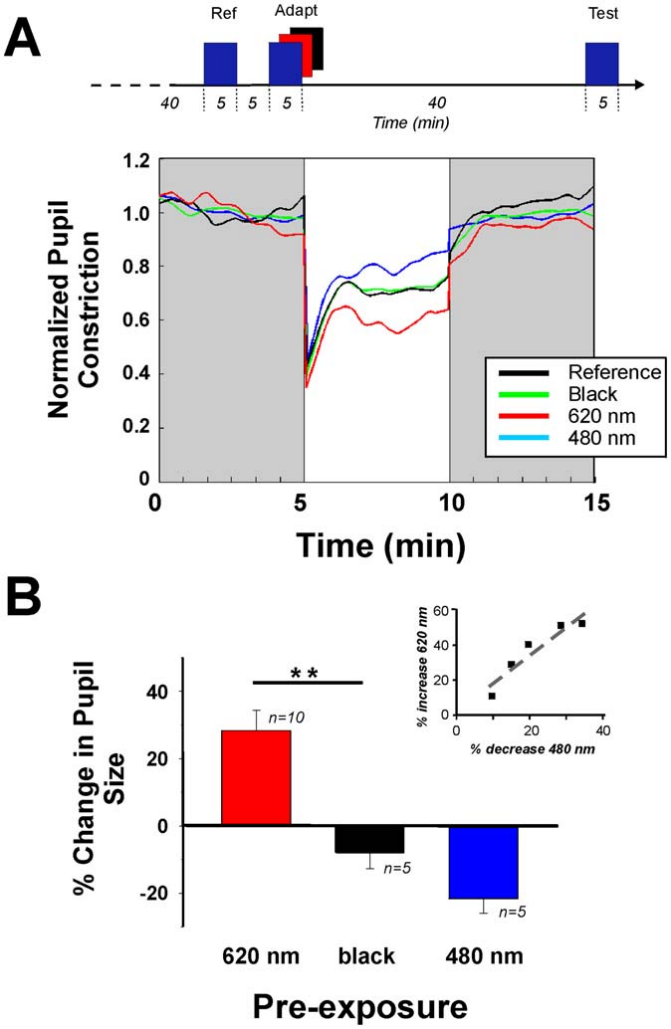
Alteration of the steady state equilibrium pupillary reflex response following pre-exposure with monochromatic lights. (A) Representative responses of a subject during the 5 minutes of 480 nm monochromatic light (test stimulation, 1e12 photons/cm^2^/sec, 5 min duration) after pre-exposures to 5 minutes to an adapting 620 nm (red line), 480 nm (blue line) or no light (“black”, green line) compared to the normalized dark adapted reference response (black line; see temporal sequence of exposure above, further details in Methods). While intervening darkness (dark control) has no effect on the subsequent pupillary response to blue light (−7.58%), the amplitude of the steady state equilibrium obtained is increased by 51.71% following a prior long wavelength exposure but is decreased by 34.46% following prior short wavelength exposure. Note that the initial transient pupillary constriction is unaffected by the different pre-exposure conditions (0–10 sec, 480 nm = −6.47±3.38%, 620 nm = 1.01±3.23%; ns) while the persistent pupillary constriction during the first minute following stimulus extinction shows a similar tendency to that observed for the steady state equilibrium. (B) Histogram summarizing the alteration of the steady state equilibrium pupil responses. Dark exposure between the reference and test pulses does not significantly alter the pupillary response (−7.97±4.70%, CI 95% = [−26.4;10.45]). In contrast, prior red (620 nm) light exposure leads to a significant increase in pupillary constriction compared to dark (28.20±6.08%, p = 0.0018, ANOVA and Newman Keuls post hoc test) while prior blue (480 nm) light exposure does not alter pupillary constriction compared to dark (−21.44±4.47%, p = 0.18). Note, as shown in the inset, that although the degree of change in responsiveness following short and long wavelength pre-exposures varied between subjects, for each individual the increase in the response following red pre-exposure is proportional to the decrease in the response following blue light pre-exposure and is significantly correlated across subjects (R^2^ = 0.88).

We then tested the hypothesis that prior light exposure would alter the steady state equilibrium PLR in a wavelength-dependent manner that is consistent with the response properties of bistable IRPs [9], [12]. Subjects received two identical 5 min exposures to 480 nm light with or without an additional adapting exposure in the intervening 40 min period of darkness (further detail in Methods). The reference exposure is designed to initially drive the photopigment system to an identical state of photoequilibrium at the beginning of each series of stimulations, prior to the adapting stimulus that drives the responsiveness of the system to a new state, assayed during the test stimulation [8], [9]. The 40 min interval after the adapting stimulus is required to allow return full recovery of rods and cones and return to the baseline condition. Prior exposure to an adapting long wavelength red light (620 nm) causes a significant increase in the steady-state equilibrium response of 28.20±6.08% whereas a prior blue (480 nm) light exposure decreases responsiveness by 21.44±4.47% (**figure 3A–B**). The dark control condition has no significant effect on the amplitude of pupil constriction. Furthermore, although the degree of change in responsiveness following short and long wavelength pre-exposures varied between subjects, there was a significant correlation across subjects for the change of response amplitude at 620 nm versus 480 nm (**figure 3B** **inset**).

**Figure 4:**
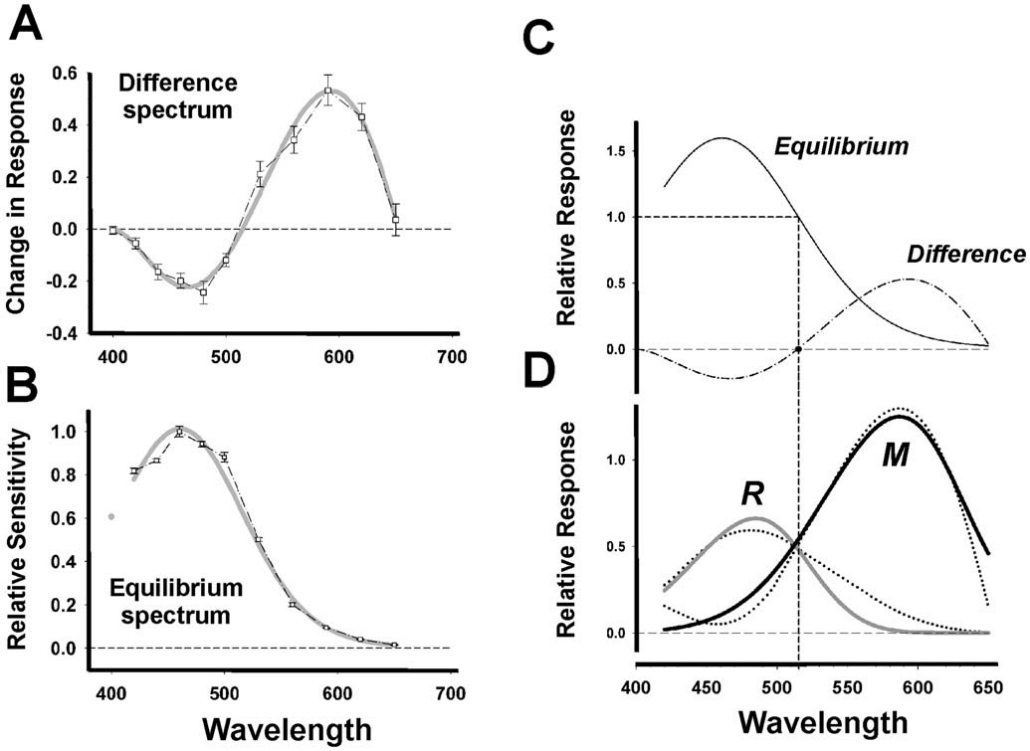
Difference and Equilibrium spectra and modelling of the putative R and M absorption spectra. (A) The difference spectrum was obtained by measuring the change in pupil constriction between the two identical reference and test stimulations (480 nm, 1e12 photons/cm^2^/sec, 5 min duration) subsequent to a 5 min pre-exposure period for 11 different wavelengths (n = 4 subjects; iso-irradiance of 14.1±0.2 log photons/cm^2^/s). Consistent with typical difference spectra of invertebrates, the response function (5-degree polynomial, R^2^ = 0.91) shows two domains: a decrease in the response between 400–515 nm (min = 466 nm) and an increase in constriction for longer wavelengths (515–650 nm, max = 592 nm). The response function also shows an isosbestic point that corresponds to the wavelength at which the pre-exposure stimulation has a neutral effect (514.3±1.2 nm). (B) Equilibrium spectrum (4-degree polynomial, R^2^ = 0.99) obtained using the pupil constriction observed in response to the different pre-exposure wavelengths (n = 4, for details see Methods). The data point at 400 nm (gray circle) is from a single subject and thus was not included in the spectral response curve. The peak response is at 460 nm. For A–B the gray line shows the fitted curve. (C) Illustrates normalization of the equilibrium spectrum (B) to 1.0 at the isosbestic point obtained from the difference spectrum (A). We applied the mathematical model developed by Stavenga [19], [20] that allows calculation of the R and M absorption spectra for IRP. (D) Illustrates the two putative spectra that peak at 481 nm (R state) and 587 nm (M state) corresponding to melanopsin and “metamelanopsin” isoforms. The two derived spectra (dashed lines) show a good fit with the vitamin A nomograms (solid gray and black lines) with maxima at 484 nm (R^2^ = 0.82) and 587 (R^2^ = 0.97) nm respectively.

We then explored the full wavelength dependence of this response alteration across the visible spectrum (400–650 nm). In IRPs, response amplitude either increases or decreases depending on the amount of photoconversion between the two *11-cis* and *all-trans retinal* bound photopigment (R and M) states generated by prior light conditions. The resulting response function describes the difference spectrum, a fundamental property of bistable photopigment systems [9], [19]. Following our exposure paradigm, we obtain a response function that reveals a region of increased responsiveness subsequent to long wavelength light exposures (>515 nm) and a region of decreased responsiveness after short wavelength lights (<515 nm, **figure 4A**). The shape of this function is typical for invertebrate photopigment difference spectra and closely resembles that obtained for fly or moth rhodopsin (see [19], [20], [31]. A critical feature of the difference spectrum is the existence of a unique wavelength at which prior light stimulation has no influence on subsequent responsiveness (zero crossing point). This “isosbestic” point represents the wavelength producing an equivalent rate of conversion between the two interconvertible R and M states of a bistable pigment system. The presence of a unique isosbestic point is strong evidence for a single bistable pigment system and despite the inter-individual differences in response amplitudes all subjects had a virtually identical isosbestic wavelength (514.3±1.2 nm).

Characterisation of a bistable photosystem also requires definition of the photoequilibrium spectrum, a function that depends on the fractions of the two R and M isoforms [9], [19], [20]. The photoequilibrium spectrum (as the difference spectrum) is a measure that reflects the response capacity of the photopigment system as a whole and not a specific photopigment state, in contrast to a rod or cone opsin action spectrum derived from the absorbance of the single *11-cis retinal* bound opsin. We propose that the steady-state equilibrium response of the pupil (see **figure 4B**) represents the equilibrium spectrum of melanopsin (i.e., the bistable photopigment equivalent of an “action spectrum”). This response spectrum shows peak sensitivity near 460 nm, but compared to a typical photopigment nomogram, shows higher than expected values in the shorter and longer wavelength regions of the spectrum.

### Modelling the underlying R-M states of the melanopsin photopigment system

We then used the photoequilibrium and difference spectra to derive the underlying R and M absorption spectra according to previous invertebrate photopigment models [9], [19]. The model is constrained by the vitamin A nomograms of the underlying R and M absorption states that combine to shape the response profiles of the photoequilibrium and difference spectra (for further explanation see Methods). The model yields two spectral response functions (**figure 4C–D**) each showing a close correspondence with the Lamb vitamin A-based photopigment nomogram. The first profile peaking at 481 nm in the blue region corresponds to the putative *11-cis retinal* bound (“R”) form of melanopsin while the second curve shows a peak in the orange region of the spectrum (587 nm) and represents the derived absorption spectrum of the *all-trans retinal* bound “M” or “meta-melanopsin” state of the photopigment. The distance between the two peaks (∼100 nm) is in the range observed for IRPs such as fly rhodopsins (R = 480 nm, M = 570–580 nm; see table I in [9]).

## Discussion

A key outcome of our study is that the obtained and derived spectral response functions of melanopsin are consistent with the principles of bistable IRP photoresponses. The photic response of the system can be driven to a stable state (here measured as the photoequilibrium of pupil constriction) that is modulated by and can be predicted from the parameters of prior light exposure. Analysis of the equilibrium and difference spectra using invertebrate photopigment models defines the two underlying photopigment absorption states of melanopsin. Our calculated peak of the *11-cis* form (R form) of melanopsin precisely matches available *in vitro* spectral absorption measures of purified amphioxus and vertebrate melanopsins [11], [25]. These values are also in close agreement with data recently obtained by Walker et al [32] in immuno-isolated mouse mRGCs (500 nm). A direct physiological or photochemical measure of the meta-melanopsin spectrum (*all-trans bound* M state) will require further *in vitro* confirmation, involving suitable purification of the functional protein, expression of stable photoproducts, and/or low-temperature spectroscopy [11], [32] but, as for IRPs in general, may be difficult to achieve due to overlap of the two R and M spectra that prevents driving the system to a pure M state [9], [33]. Our results nevertheless concur with all previous *in vitro* studies that have demonstrated that long wavelength light, greater than the isosbestic point of 515 nm here defined, is effective to drive melanopsin from the *all-trans* to an *11-cis* bound form [4], [5], [10], [11], [13]. An ancillary, but as yet unresolved, question is the possible existence of a light-independent pathway for “dark” reversion of the *all-trans* isoform back to the *11-cis* isoform that is present in certain IRPs [9]. Recent experimental results are controversial since Walker et al. [9] have provided evidence in support of, but Melyan et al. [4] and Giesbers et al. [33] against, the presence of a light-independent recovery mechanism. Nevertheless, our results would suggest that if dark regeneration occurs, this does not seem to be apparent within the 45 minute interval used in the present experimental conditions.

Our findings on the spectral sensitivity of the melanopsin photopigment system also reconcile previous results obtained in humans and in animals by emphasizing the importance of the temporal features of the melanopsin system response to light. The spectral sensitivity of the steady state equilibrium pupil response (460 nm) is in agreement with studies that have used prolonged light exposures such as melatonin suppression assays [34], [35]. These studies report a maximal response in the short wavelength region (459–464 nm) but for which it is debatable whether a single opsin nomogram template optimally describes the data. In contrast, measures of several physiological responses using short duration light exposures (generally less than 10 sec) report a peak sensitivity for melanopsin between 476–484 nm, based on pupil and mRGCs recordings in mouse [26], [27], [36] in primate [15], [28], and for pupil sensitivity in a single blind human considered to lack functional rods and cones [37]. The short duration light exposures may not optimally allow the photopigment system to achieve a stable state of photoequilibrium, perhaps related to the long latency photic response of melanopsin. In further support of this, recent reports by McDougal and Gamlin [38], [39] indicate that for long exposures of 30 or 100 sec, the peak sensitivity of the sustained pupil constriction response is shifted to shorter wavelengths (471 nm) compared to the broad peak ∼510 nm stated in their previous report [15] associated with a log unit higher than expected values for wavelengths >550 nm compared to a Vitamin A nomogram. These results are thus coherent with our findings of a peak response at 460 nm for exposures of still longer durations between 180–300 sec required to obtain a steady-state equilibrium. Since in humans synaptic blockade of outer retinal photoreceptors is not feasible, it cannot be totally excluded that the response of the steady-state pupillary constriction contains some contribution of rods and cones. However, several findings argue against this. Mice lacking melanopsin fail to sustain pupillary constriction after 30 sec of bright light exposure [17] indicating that functional rods and cones alone are incapable of maintaining the response. Elimination of rod and cone inputs using synaptic blockers in mice does not significantly alter the sustained pupillary constriction compared to intact animals, whereas the addition of a specific TRPC channel blocker of the melanopsin phototransduction pathway completely abolishes sustained pupillary constriction to light [40]. In the absence of melanopsin, neuronal responses in mice do not express either sustained responses to light or response enhancement by prior long wavelength stimulations [12]. In addition, modelling the equilibrium and difference spectra accurately predicts the known peak and spectral response nomogram function of purified, expressed melanopsin [3], [10], [11], [25]. The possible involvement of a subpopulation of melanopsin expressing cones in the steady state and post-stimulus responses of the pupil is also possible [41].

Furthermore, our results suggest a form of rapid, reversible “photic memory” since information of the prior spectral light conditions, here occurring nearly 1 hour earlier, is retained and shapes the subsequent response to light. These results emphasize the importance of previous light history in chronobiology [42] and raise the possibility that appropriate manipulation of spectral light composition in industrial, domestic and in clinical phototherapy applications can be exploited to manipulate the response capacity of melanopsin-dependent circadian and other non-visual responses. Since ocular pathologies such as glaucoma cause degeneration of melanopsin RGCs [43] this may alter not only pupil responses [44] but also other non-visual responses to light.

**Figure 5:**
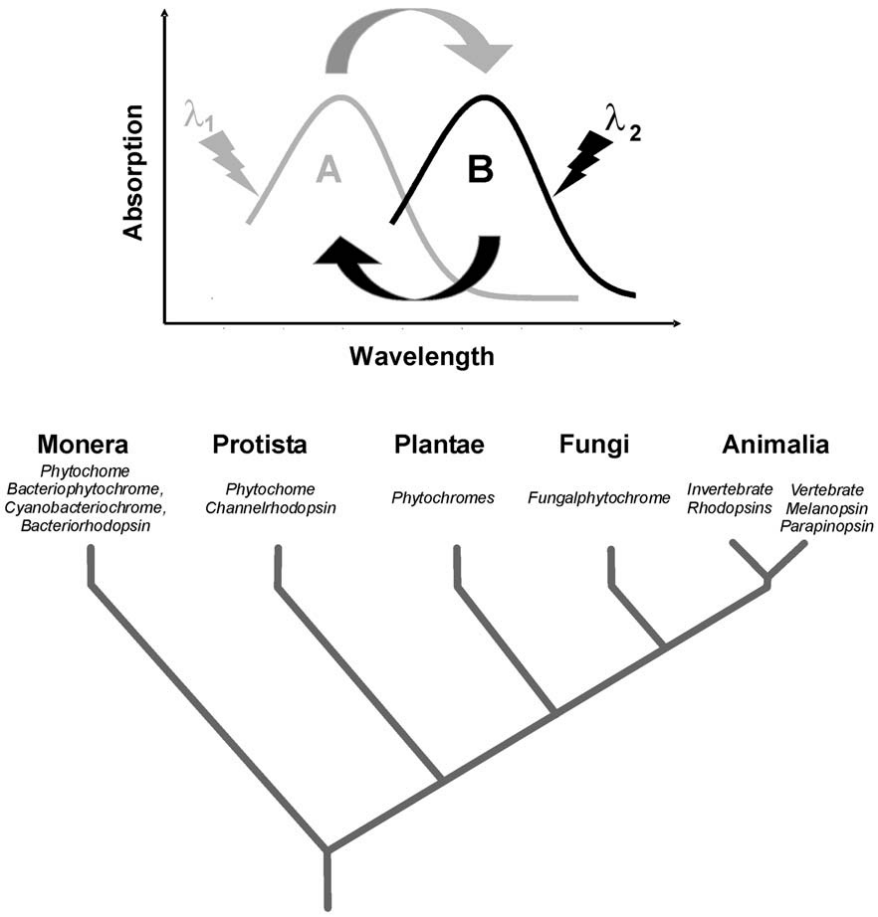
Distribution in living organisms according to kingdom of photopigments that act as photosensory photo-switches. In bistable pigments different wavelengths of light (λ_1_, λ_2_) trigger a conformational change between two spectrally distinct, relatively stable isoforms (A–B, above): an inactive form and an active form that elicits physiological functions. Many different bistable photopigment classes that use different chromophores (see Table S1) are present in living organisms, while Melanopsin has only been described in vertebrate species and in the chordate *Amphioxus*. Parapinopsin is a UV sensitive bistable pigment present in lamprey pineal (see Table S1 for further details).

The presence of a functional bistable mechanism in humans additionally highlights a ubiquitous feature of photoswitchable photosensors across living organisms, including bacteria (Bacteriophytochrome), cyanobacteria (Cyanobacteriochrome), fungi (Fungalphytochromes), plants (Phytochromes), invertebrates (IRPs) and vertebrates (melanopsin; **figure 5** **, Table S1**).

## Supporting Information

Figure S1 Action spectra during different time windows of the early phases of the pupillary response. The sensitivity curve of the initial 0–30 seconds response to light (see figure 2) is very similar to sensitivity curves analyzed during other early, mid or later temporal periods of the initial response to light (0–5 sec, 5–10 sec and 5–30 sec). The response profiles are also similar to the previous descriptions of the “tonic response” recorded in monkey (5–10 sec, [1]) and in human (0–6 sec, [2]). 1. Gamlin PD, McDougal DH, Pokorny J, Smith VC, Yau KW, et al. (2007) Human and macaque pupil responses driven by melanopsin-containing retinal ganglion cells. Vision Res 47: 946–954. 2. Kimura E, Young RS (1995) Nature of the pupillary responses evoked by chromatic flashes on a white background. Vision Res 35: 897–906.
(2.37 MB TIF)

Figure S2 Long duration persistence of post-stimulus pupil constriction. After the extinction of the light, the pupil does not redilate immediately to the dark adapted baseline level. Persistence of pupil constriction depends on the wavelength and the irradiance of the preceding light stimulus. The example shown here compares the time for the pupil to return to the dark adapted state following 5 min exposure to 480 and 620 nm monochromatic light at different irradiances (3–4 exposures for each irradiance in 2 subjects). Following 480 nm at irradiances >1e14 photons/cm^2^/sec the pupil constriction has still not returned to the baseline at the end of the recording period, 5 minutes after light extinction (hence the absence of error bars). This persistence of pupil constriction in humans is analogous to the short wavelength-triggered pupil response in flies attributed to the prolonged depolarizing after-potential (PDA; [1]). 1. Hillman P, Hochstein S, Minke B (1983) Transduction in invertebrate photoreceptors: role of pigment bistability. Physiol Rev 63: 668–772.
(3.41 MB TIF)

Table S1 Distribution, chromophore and functions of bistable photopigments in living organisms. Different photopigment classes utilize specific light-sensitive chromophores, mainly retinal (vitamin A) in the rhodopsins and bilins in the phytochromes. Bistable photopigments are involved in a wide range of biological functions including perceptual vision and non-visual light detection. Many organisms also express other photopigments that require non-light dependent biochemical mechanisms for regeneration of the chromophore (rods, cones, cryptochromes, phototropins, etc…). 1. Altimus CM, Guler AD, Villa KL, McNeill DS, Legates TA, et al. (2008) Rods-cones and melanopsin detect light and dark to modulate sleep independent of image formation. Proc Natl Acad Sci U S A 105: 19998–20003. 2. Cajochen C, Munch M, Kobialka S, Krauchi K, Steiner R, et al. (2005) High sensitivity of human melatonin, alertness, thermoregulation, and heart rate to short wavelength light. J Clin Endocrinol Metab 90: 1311–1316. 3. Vandewalle G, Schmidt C, Albouy G, Sterpenich V, Darsaud A, et al. (2007) Brain responses to violet, blue, and green monochromatic light exposures in humans: prominent role of blue light and the brainstem. PLoS ONE 2: e1247. 4. Koyanagi M, Kawano E, Kinugaw E Oish T, Shichida, Y et al. (2004) Bistable UV pigment in the lamprey pineal. Proc Natl Acad Sci U S A 101: 6687–6691. 5. Montgomery BL (2007) Sensing the light: photoreceptive systems and signal transduction in cyanobacteria. Mol Microbiol 64: 16–27. 6. Sineshchekov OA, Govorunova EG (2001) Rhodopsin receptors of phototaxis in green flagellate algae. Biochemistry (Mosc) 66: 1300–1310. 7. Vogeley L, Sineshchekov OA, Trivedi VD, Sasaki J, Spudich JL, et al. (2004) Anabaena sensory rhodopsin: a photochromic color sensor at 2.0 A. Science 306: 1390–1393. 8. Nagel G, Szellas T, Kateriya S, Adeishvili N, Hegemann P, et al. (2005) Channelrhodopsins: directly light-gated cation channels. Biochem Soc Trans 33: 863–866. 9. Lamparter T (2004) Evolution of cyanobacterial and plant phytochromes. FEBS Lett 573: 1–5. 10. Lariguet P, Dunand C (2005) Plant photoreceptors: phylogenetic overview. J Mol Evol 61: 559–569. 11. Sharrock RA (2008) The phytochrome red/far-red photoreceptor superfamily. Genome Biol 9: 230. 12. Falciatore A, Bowler C (2005) The evolution and function of blue and red light photoreceptors. Curr Top Dev Biol 68: 317–350. 13. Wang H (2005) Signaling mechanisms of higher plant photoreceptors: a structure-function perspective. Curr Top Dev Biol 68: 227–261. 14. Vierstra RD, Davis SJ (2000) Bacteriophytochromes: new tools for understanding phytochrome signal transduction. Semin Cell Dev Biol 11: 511–521.
(0.07 MB DOC)
