# Supplementary figures and images for: Melanopsin Bistability: A Fly's Eye Technology in the Human Retina
Source: PLoS One. 2009 Jun 24;4(6):e5991. doi: 10.1371/journal.pone.0005991 (PMC2695781; doi:10.1371/journal.pone.0005991)

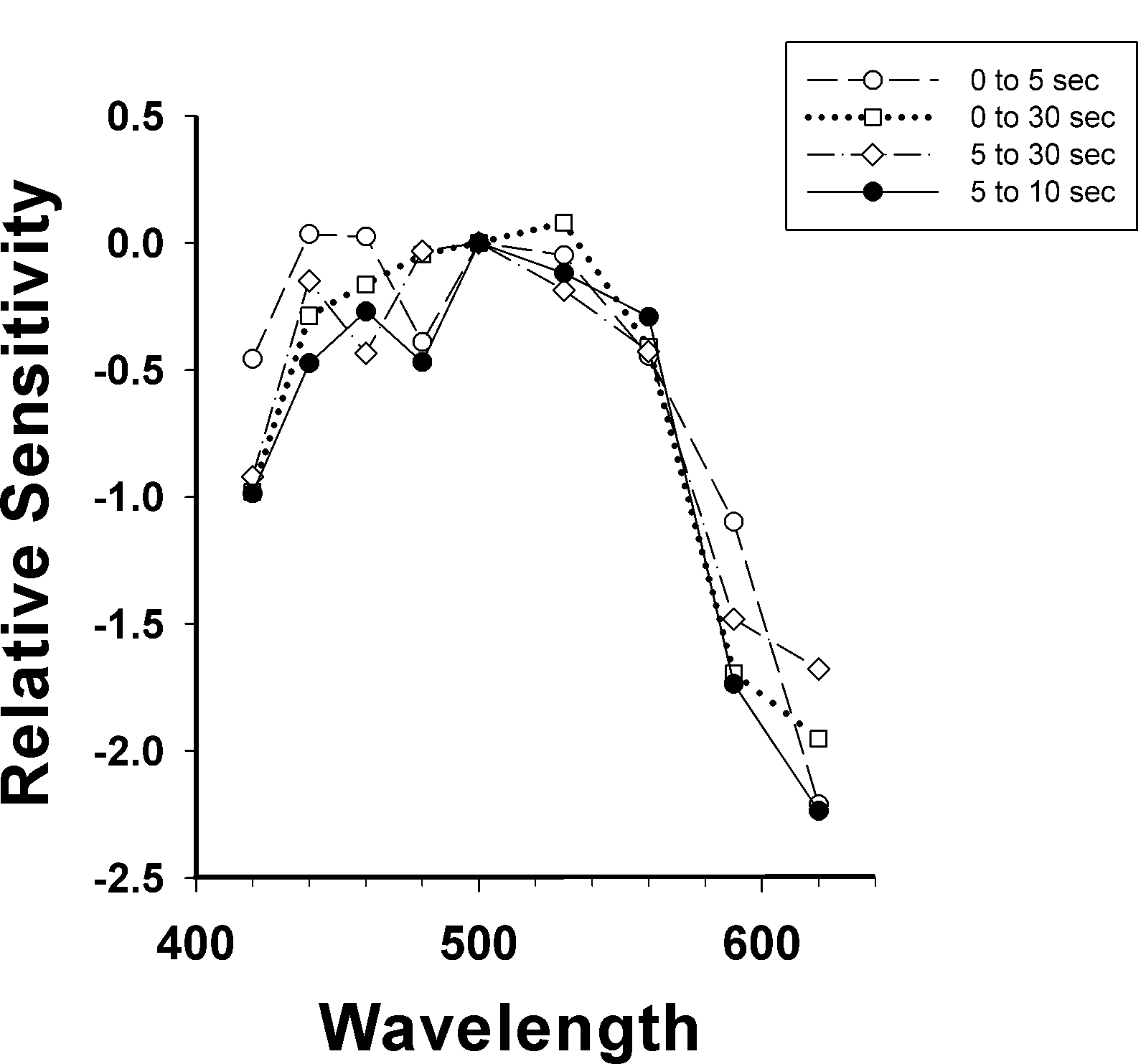

Supplement: Figure S1 — Action spectra during different time windows of the early phases of the pupillary response. The sensitivity curve of the initial 0–30 seconds response to light (see figure 2) is very similar to sensitivity curves analyzed during other early, mid or later temporal periods of the initial response to light (0–5 sec, 5–10 sec and 5–30 sec). The response profiles are also similar to the previous descriptions of the “tonic response” recorded in monkey (5–10 sec, [1]) and in human (0–6 sec, [2]). 1. Gamlin PD, McDougal DH, Pokorny J, Smith VC, Yau KW, et al. (2007) Human and macaque pupil responses driven by melanopsin-containing retinal ganglion cells. Vision Res 47: 946–954. 2. Kimura E, Young RS (1995) Nature of the pupillary responses evoked by chromatic flashes on a white background. Vision Res 35: 897–906. (2.37 MB TIF) [file pone.0005991.s001.tif]

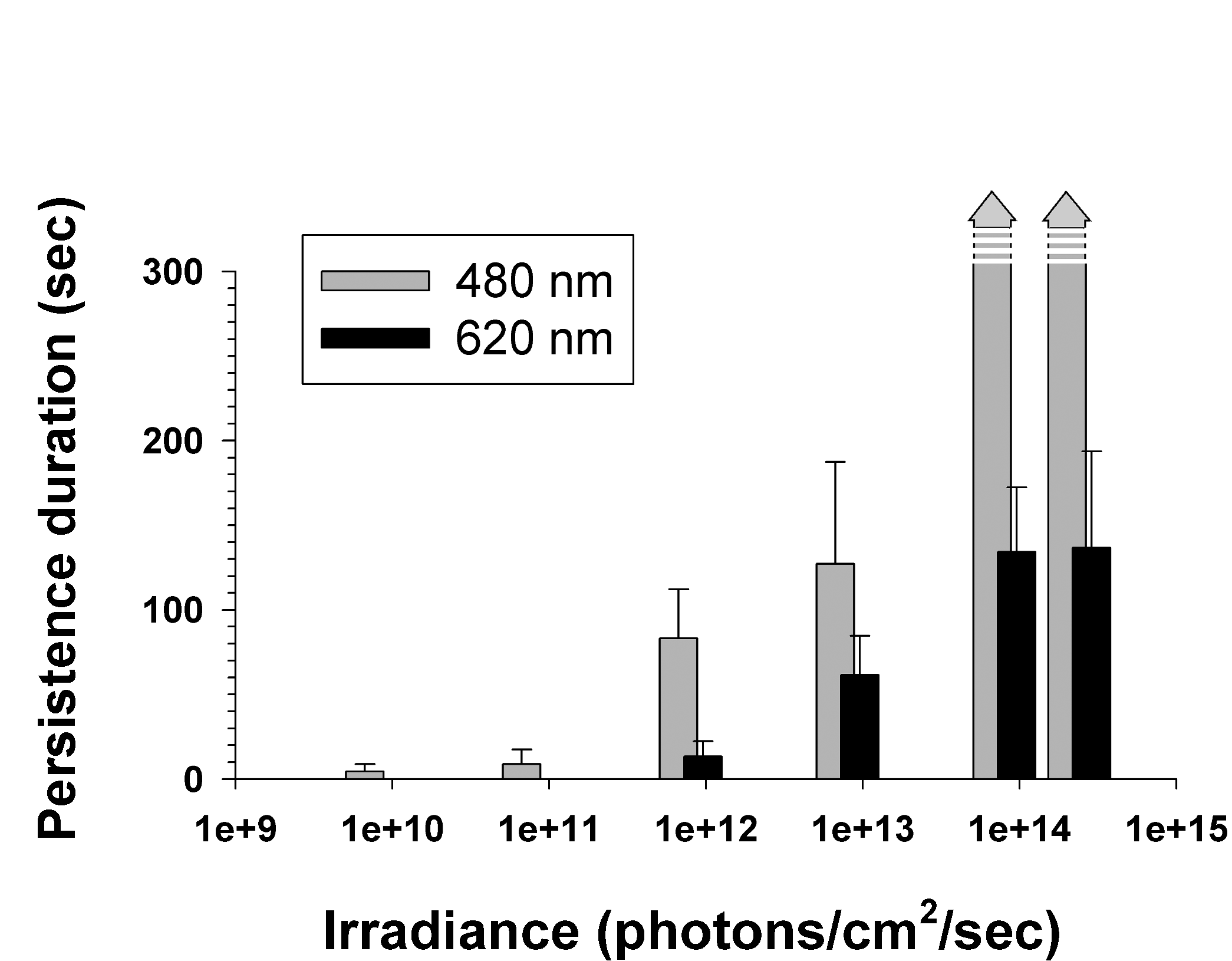

Supplement: Figure S2 — Long duration persistence of post-stimulus pupil constriction. After the extinction of the light, the pupil does not redilate immediately to the dark adapted baseline level. Persistence of pupil constriction depends on the wavelength and the irradiance of the preceding light stimulus. The example shown here compares the time for the pupil to return to the dark adapted state following 5 min exposure to 480 and 620 nm monochromatic light at different irradiances (3–4 exposures for each irradiance in 2 subjects). Following 480 nm at irradiances >1e14 photons/cm2/sec the pupil constriction has still not returned to the baseline at the end of the recording period, 5 minutes after light extinction (hence the absence of error bars). This persistence of pupil constriction in humans is analogous to the short wavelength-triggered pupil response in flies attributed to the prolonged depolarizing after-potential (PDA; [1]). 1. Hillman P, Hochstein S, Minke B (1983) Transduction in invertebrate photoreceptors: role of pigment bistability. Physiol Rev 63: 668–772. (3.41 MB TIF) [file pone.0005991.s002.tif]
