# Supplementary material for: Melanopsin Bistability: A Fly's Eye Technology in the Human Retina
Source: PLoS One. 2009 Jun 24;4(6):e5991. doi: 10.1371/journal.pone.0005991 (PMC2695781; doi:10.1371/journal.pone.0005991)
Supplement: Table S1 — Distribution, chromophore and functions of bistable photopigments in living organisms. Different photopigment classes utilize specific light-sensitive chromophores, mainly retinal (vitamin A) in the rhodopsins and bilins in the phytochromes. Bistable photopigments are involved in a wide range of biological functions including perceptual vision and non-visual light detection. Many organisms also express other photopigments that require non-light dependent biochemical mechanisms for regeneration of the chromophore (rods, cones, cryptochromes, phototropins, etc…). 1. Altimus CM, Guler AD, Villa KL, McNeill DS, Legates TA, et al. (2008) Rods-cones and melanopsin detect light and dark to modulate sleep independent of image formation. Proc Natl Acad Sci U S A 105: 19998–20003. 2. Cajochen C, Munch M, Kobialka S, Krauchi K, Steiner R, et al. (2005) High sensitivity of human melatonin, alertness, thermoregulation, and heart rate to short wavelength light. J Clin Endocrinol Metab 90: 1311–1316. 3. Vandewalle G, Schmidt C, Albouy G, Sterpenich V, Darsaud A, et al. (2007) Brain responses to violet, blue, and green monochromatic light exposures in humans: prominent role of blue light and the brainstem. PLoS ONE 2: e1247. 4. Koyanagi M, Kawano E, Kinugaw E Oish T, Shichida, Y et al. (2004) Bistable UV pigment in the lamprey pineal. Proc Natl Acad Sci U S A 101: 6687–6691. 5. Montgomery BL (2007) Sensing the light: photoreceptive systems and signal transduction in cyanobacteria. Mol Microbiol 64: 16–27. 6. Sineshchekov OA, Govorunova EG (2001) Rhodopsin receptors of phototaxis in green flagellate algae. Biochemistry (Mosc) 66: 1300–1310. 7. Vogeley L, Sineshchekov OA, Trivedi VD, Sasaki J, Spudich JL, et al. (2004) Anabaena sensory rhodopsin: a photochromic color sensor at 2.0 A. Science 306: 1390–1393. 8. Nagel G, Szellas T, Kateriya S, Adeishvili N, Hegemann P, et al. (2005) Channelrhodopsins: directly light-gated cation channels. Biochem Soc Trans 33: 863–866. 9. Lamparter T ( [file pone.0005991.s003.doc]

| **Photopigment Family** | **Organisms** | **Chromophore** | **Isoform Spectral domains** (**λ**_1_, **λ**_2_) | **Biological Functions** |
| --- | --- | --- | --- | --- |
| **Melanopsin** | **Vertebrates** (+amphioxus) | *retinal* | Blue-orange | pupil constriction, IpRGC responses, SCN neuron responses, photic phase shifts, circadian photoreception, alertness, sleep quality (see references in main manuscript + [1-3]) |
| **Parapinopsin** | **Vertebrates** | *retinal_2_* | UV-green | lamprey pineal, photoperiodic responses [4] |
| **Rhodopsins** | **Invertebrates**  **Cyanobacteria**  bacteriorhodopsin  **Green algae**  channelrhodopsin | *retinal*  *retinal*  *retinal* | Several domains from UV-Red  Green-orange  Blue-orange | all dependent physiology including vision, phototaxis, circadian photoreception, etc.  phototropism [5-7]  phototropism [8] |
| **Phytochrome** | **Plants**  **Green algae** | *phytochromobilin*  *phytochromobilin* | Red-far red  Red-far red | metabolic responses to light, photomorphogenesis, hypocotyl, elongation, shade-avoidance, seed germination, flowering, circadian rhythms  phototropism, chloroplast relocation, stomata opening, sexual development [9-13] |
| **Cyanobacteriochrome** | **Cyanobacteria** | *phycocyanobilin* | Red-far red | control of phototaxis, cell growth and differentiation, metabolism, chromatic adaptation [5,10,12] |
| **Bacteriophytochrome** | **Bacteria** | *biliverdin* | Red-far red | chromatic adaptation, phototropism [11,12,14] |
| **Fungalphytochrome** | **Fungi** | *biliverdin* | Red-far red | sexual -asexual reproduction, sporulation, mycotoxin development |
